# A comprehensive study on size and definition of the core group in the proven and young algorithm for single-step GBLUP

**DOI:** 10.1186/s12711-022-00726-6

**Published:** 2022-05-20

**Authors:** Rostam Abdollahi-Arpanahi, Daniela Lourenco, Ignacy Misztal

**Affiliations:** Department of Animal and Dairy Science, University of Georgia, Athens, GA 30602 USA

## Abstract

**Background:**

The algorithm for proven and young (APY) has been suggested as a solution for recursively computing a sparse representation for the inverse of a large genomic relationship matrix (**G**). In APY, a subset of genotyped individuals is used as the core and the remaining genotyped individuals are used as noncore. Size and definition of the core are relevant research subjects for the application of APY, especially given the ever-increasing number of genotyped individuals.

**Methods:**

The aim of this study was to investigate several core definitions, including the most popular animals (MPA) (i.e., animals with high contributions to the genetic pool), the least popular males (LPM), the least popular females (LPF), a random set (Rnd), animals evenly distributed across genealogical paths (Ped), unrelated individuals (Unrel), or based on within-family selection (Fam), or on decomposition of the gene content matrix (QR). Each definition was evaluated for six core sizes based on prediction accuracy of single-step genomic best linear unbiased prediction (ssGBLUP) with APY. Prediction accuracy of ssGBLUP with the full inverse of **G** was used as the baseline. The dataset consisted of 357k pedigreed Duroc pigs with 111k pigs with genotypes and ~ 220k phenotypic records.

**Results:**

When the core size was equal to the number of largest eigenvalues explaining 50% of the variation of **G** (n = 160), MPA and Ped core definitions delivered the highest average prediction accuracies (~ 0.41−0.53). As the core size increased to the number of eigenvalues explaining 99% of the variation in **G** (n = 7320), prediction accuracy was nearly identical for all core types and correlations with genomic estimated breeding values (GEBV) from ssGBLUP with the full inversion of **G** were greater than 0.99 for all core definitions. Cores that represent all generations, such as Rnd, Ped, Fam, and Unrel, were grouped together in the hierarchical clustering of GEBV.

**Conclusions:**

For small core sizes, the definition of the core matters; however, as the size of the core reaches an optimal value equal to the number of largest eigenvalues explaining 99% of the variation of **G**, the definition of the core becomes arbitrary.

## Background

Single-step genomic best linear unbiased prediction (ssGBLUP) has been adopted as a standard for genomic evaluation in many livestock species. ssGBLUP is an extended version of the conventional BLUP that integrates the inverses of the genomic ($\mathbf{G}$) and pedigree ($\mathbf{A}$) relationship matrices into a new matrix represented by ${\mathbf{H}}^{-1}$ [1]. Matrix inversion has a cubic cost; therefore, inverting $\mathbf{G}$ for more than 100K genotyped individuals is a barrier for the application of ssGBLUP [2]. Several approaches have been proposed to resolve this issue [3–5]. Faux et al. [3] attempted to approximate the inverse of $\mathbf{G}$ with incomplete Cholesky factorization. However, the approximation was not accurate enough and the steps suggested to increase accuracy were time-demanding. Liu et al. [5] proposed the single-step single nucleotide polymorphism (SNP)-BLUP, but there was a lack of convergence with real data. Fernando et al. [4] developed a version of single-step SNP-BLUP in the Bayesian framework. Although convergence rates were satisfactory, imputation of genotypes for non-genotyped animals requires large memory for big populations, and computations need graphics processing units (GPU) for efficient parallelization. However, these drawbacks were resolved by Fernando et al. [6] and the single-step SNP-BLUP approach has been implemented for genomic evaluation with large genotype data sets. In addition, Vandenplas et al. [7] proposed an improved preconditioner to accelerate the convergence rate in single-step SNP-BLUP models, making them feasible for large-scale genomic evaluations.

Misztal et al. [8] proposed an algorithm called “algorithm for proven and young” (APY) based on genomic recursions to create a sparse representation of the inverse of $\mathbf{G}$. In APY, genotyped animals are partitioned into two groups, representing core and noncore animals. Genomic estimated breeding values (GEBV) of noncore animals are conditioned on the GEBV of core animals. The cost of computing ${\mathbf{G}}^{-1}$ based on APY is cubic to the number of core animals and linear to the number of noncore animals [8]. The core size depends on the dimensionality of $\mathbf{G}$, which has been addressed by the eigenvalue decomposition of $\mathbf{G}$ [9]; the dimensionality is a function of effective population size and genome length [10]. However, the choice of core animals is of interest, and there is a question on whether a pre-defined choice (e.g., based on the amount of information available for the animals or their impact on the population) would allow a further reduction in the number of core animals.

In a seminal study, Misztal [11] hypothesized that the accuracy of APY depends on how well animals in the core group represent the independent chromosome segments (ICS) that are present in the population. Several studies have investigated the core definition in APY ssGBLUP [2, 12–17]. Some have reported that, in cattle, using proven bulls as core led to nearly identical accuracy as using randomly selected core sets [2, 13, 18]. However, in pigs and sheep, several studies have reported different EBV and accuracies of prediction by different core definitions [14, 16, 17]. In all these studies, the number of genotyped animals per breed was less than 22k, and none investigated the interplay between core size and definition, especially for larger genotyped populations. Therefore, the aim of this study was to assess the performance of eight core definitions for six core sizes based on the eigenvalue decomposition of $\mathbf{G}$, on the prediction accuracy of ssGBLUP using 111k genotyped Duroc pigs.

## Methods

### Data structure

We used a dataset collected from 2012 to 2021 that consisted of 357,220 pedigreed Duroc pigs, of which 111,583 were genotyped for 32,824 SNPs after quality control. The studied traits included average daily gain (ADG) from birth to the end of the nursery period at 11 weeks of age (ADGn, N = 221,955), ADG during the finishing period until 23 weeks of age (ADGf, N = 195,946), and backfat (BF, N = 195,967). Descriptive statistics of the studied traits are in Table 1. The proportion of genotyped animals increased over time from 8% for pigs born in 2012 to 42% for pigs born in 2020. All males born from 2017 to 2020 at 11 weeks of age were genotyped but only a subset of females, selected based on body weight, was genotyped during this period. This population has been under genomic selection since 2018 [19].

**Table 1:** Descriptive statistics of the data and estimates of heritability used in the analyses

Trait	Number of records	Mean	SD	Heritability
ADGn (g)	221,955	347.50	64.36	0.20
ADGf (g)	195,946	959.92	123.62	0.21
BF (mm)	195,967	10.39	2.56	0.34

*SD* standard deviation, *ADGn* average daily gain in nursery, *ADGf* average daily gain in finishing, *BF* backfat

### Statistical analyses

The statistical model used for genetic evaluation in a multiple-trait animal model framework was:

1 $${\mathbf{y}}_{\mathrm{t}}={\mathbf{X}\mathbf{b}}_{\mathrm{t}}+{\mathbf{W}}_{1}{\mathbf{l}}_{\mathrm{t}}+{\mathbf{W}}_{2}{\mathbf{p}\mathbf{e}}_{\mathrm{t}}+{\mathbf{W}}_{3}{\mathbf{a}}_{\mathrm{t}}+{\mathbf{e}}_{\mathrm{t}},$$

where ${\mathbf{y}}_{\mathrm{t}}$ is the vector of observations for trait $t$, which refers to ADGn, ADGf, or BF; ${\mathbf{b}}_{\mathrm{t}}$ is the vector of fixed effects; ${\mathbf{l}}_{\mathrm{t}}$ (45,185 levels), ${\mathbf{p}\mathbf{e}}_{\mathrm{t}}$ (9923 levels), and ${\mathbf{a}}_{\mathrm{t}}$ (357,220 animals) are the vectors of random effects for litter, pen, and of direct additive genetic effects, respectively; ${\mathbf{e}}_{\mathrm{t}}$ is the vector of residuals. $\mathbf{X}$, ${\mathbf{W}}_{1}$, ${\mathbf{W}}_{2}$, and ${\mathbf{W}}_{3}$ are design matrices for the effects in $\mathbf{b}$, ${\mathbf{l}}_{\mathrm{t}}$, ${\mathbf{p}\mathbf{e}}_{\mathrm{t}}$, and ${\mathbf{a}}_{\mathrm{t}}$, respectively. Direct additive genetic and litter effects were fit for three traits and the pen effect was fit for ADGf and BF. The fixed effects were sex for all traits, herd-year-week for ADGn, and barn for ADGn and BF. Age at final weight was considered as a covariate for ADGn and ADGf.

The (co)variance structure assumed for the random effects was:

$$\mathrm{var}\left[\begin{array}{c}\mathbf{l}\\ \mathbf{p}\mathbf{e}\\ \mathbf{a}\\ \mathbf{e}\end{array}\right]=\mathrm{var}\left[\begin{array}{c}{\mathbf{l}}_{\mathrm{ADGn}}\\ {\mathbf{l}}_{\mathrm{ADGf}}\\ {\mathbf{l}}_{\mathrm{BF}}\\ {\mathbf{p}\mathbf{e}}_{\mathrm{ADGf}}\\ {\mathbf{p}\mathbf{e}}_{\mathrm{BF}}\\ {\mathbf{a}}_{\mathrm{ADGn}}\\ {\mathbf{a}}_{\mathrm{ADGf}}\\ {\mathbf{a}}_{\mathrm{BF}}\\ \mathbf{e}\end{array}\right]=\left[\begin{array}{ccccccccc}\mathbf{I}{\upsigma }_{{\mathrm{l}}_{\mathrm{ADGn}}}^{2}& \mathbf{I}{\upsigma }_{{\mathrm{l}}_{\mathrm{ADGn},\mathrm{ADGf}}}& \mathbf{I}{\upsigma }_{{\mathrm{l}}_{\mathrm{ADGn},\mathrm{BF}}}& 0& 0& 0& 0& 0& 0\\ & \mathbf{I}{\upsigma }_{{\mathrm{l}}_{\mathrm{ADGf}}}^{2}& \mathbf{I}{\upsigma }_{{\mathrm{l}}_{\mathrm{ADGf},\mathrm{BF}}}& 0& 0& 0& 0& 0& 0\\ & & \mathbf{I}{\upsigma }_{{\mathrm{l}}_{\mathrm{BF}}}^{2}& 0& 0& 0& 0& 0& 0\\ & & & \mathbf{I}{\upsigma }_{{\mathrm{pe}}_{\mathrm{ADGf}}}^{2}& \mathbf{I}{\upsigma }_{{\mathrm{pe}}_{\mathrm{ADGf},\mathrm{BF}}}& 0& 0& 0& 0\\ & & & & \mathbf{I}{\upsigma }_{{\mathrm{pe}}_{\mathrm{BF}}}^{2}& 0& 0& 0& 0\\ & & & & & \mathbf{H}{\upsigma }_{{\mathrm{a}}_{\mathrm{ADGn}}}^{2}& \mathbf{H}{\upsigma }_{{\mathrm{a}}_{\mathrm{ADGn},\mathrm{ADGf}}}& \mathbf{H}{\upsigma }_{{\mathrm{a}}_{\mathrm{ADGn},\mathrm{BF}}}& 0\\ & & \mathrm{symmetric}& & & & \mathbf{H}{\upsigma }_{{\mathrm{a}}_{\mathrm{ADGn}}}^{2}& \mathbf{H}{\upsigma }_{{\mathrm{a}}_{\mathrm{ADGf},\mathrm{BF}}}& 0\\ & & & & & & & \mathbf{H}{\upsigma }_{{\mathrm{a}}_{\mathrm{BF}}}^{2}& 0\\ & & & & & & & & \mathbf{R}\otimes\mathbf{I}\end{array}\right],$$

where ${\upsigma }_{\mathrm{l}}^{2}$, ${\upsigma }_{\mathrm{pe}}^{2}$, and ${\upsigma }_{\mathrm{a}}^{2}$ are variances of the effects of litter size, pen, and additive genetics; ${\upsigma }_{{\mathrm{i}}_{\mathrm{j}}}$ denotes the covariance components of the $\mathrm{i}$-th effect for the $\mathrm{j}$-th combination of traits, $\mathbf{R}$ is a 3 × 3 matrix with residual (co)variance between the traits; $\mathbf{H}$ is the realized relationship matrix used in ssGBLUP, and $\mathbf{I}$ is an identity matrix with dimensions equal to the number of levels of the corresponding random effects. The (co)variance component estimates were those used in the official genetic evaluations of this population.

The relationship matrix $\mathbf{H}$ was constructed by combining relationship matrices based on pedigree ($\mathbf{A}$) and genomics ($\mathbf{G}$), following [1]. The $\mathbf{G}$ matrix was constructed as follows:

$$\mathbf{G}=\frac{\mathbf{Z}{\mathbf{Z}}^{\mathrm{^{\prime}}}}{2\sum {p}_{j}(1-{p}_{j})},$$

where $\mathbf{Z}$ is the gene content matrix coded as 0, 1, and 2 for *AA*, *AB*, and *BB* genotypes, respectively, and then centered by subtracting twice the frequency of the major allele of SNP $j$ (${p}_{j}$), following [20]. To avoid singularity problems, 95% of $\mathbf{G}$ was blended with 5% of ${\mathbf{A}}_{22}$ [20]. Genetic evaluation using ssGBLUP was conducted using the BLUPF90 family of programs [21].

### APY algorithm

Computing ${\mathbf{G}}^{-1}$, which is part of ${\mathbf{H}}^{-1}$, becomes expensive as more genotyped animals are available. Alternatively, a sparse representation of ${\mathbf{G}}^{-1}$ can be created using APY [8], in which animals are categorized as either core or noncore. A brief description of the APY algorithm is given below. See Misztal [11] for a more detailed description. In APY, the structure of $\mathbf{G}$ and ${\mathbf{G}}_{APY}^{-1}$ is as follows:

$$\mathbf{G}=\left[\begin{array}{cc}{\mathbf{G}}_{\mathrm{cc}}& {\mathbf{G}}_{\mathrm{cn}}\\ {\mathbf{G}}_{\mathrm{nc}}& {\mathbf{G}}_{\mathrm{nn}}\end{array}\right],$$

where ${\mathbf{G}}_{\mathrm{cc}}$ is the genomic covariance between animals in the core set, ${\mathbf{G}}_{\mathrm{cn}}$ is the genomic relationship between core and noncore animals and ${\mathbf{G}}_{\mathrm{nn}}$ is the genomic relationship between noncore animals. For all core definition analyses described below, ${\mathbf{G}}^{-1}$ was obtained using APY (${\mathbf{G}}_{APY}^{-1}$), with the direct inverse computed only for core animals and the elements of ${\mathbf{G}}^{-1}$ related to core and noncore animals obtained using the recursion formula presented by Misztal et al. [8]:

$${\mathbf{G}}_{APY}^{-1}=\left[\begin{array}{cc}{\mathbf{G}}_{\mathbf{c}\mathbf{c}}^{-1}& {\mathbf 0}\\ {\mathbf 0}& {\mathbf 0}\end{array}\right]+\left[\begin{array}{c}-{\mathbf{G}}_{\mathrm{cc}}^{-1}{\mathbf{G}}_{\mathrm{cn}}\\ \mathbf{I}\end{array}\right]{\mathbf{M}}_{\mathrm{nn}}^{-1}\left[\begin{array}{cc}-{\mathbf{G}}_{\mathrm{nc}}{\mathbf{G}}_{\mathrm{cc}}^{-1}& \mathbf{I}\end{array}\right],$$

where ${\mathbf{M}}_{\mathrm{nn}}$ is a diagonal matrix of genomic Mendelian sampling terms, with diagonal elements equal to:

$${m}_{ii}={g}_{ii}-{g}_{ic}{\mathbf{G}}_{cc}^{-1}{g}_{ci},$$

where ${g}_{ii}$ is the $i$th diagonal element of ${\mathbf{G}}_{\mathrm{nn}}$.

### Core sizes

The number of animals in the core (i.e. core size) was determined using the singular value decomposition of the gene content matrix $\mathbf{Z}$ [9]. The numbers of largest eigenvalues that explained 50, 80, 90, 95, and 99% of the variation of $\mathbf{G}$ represent the core sizes and were equal to 160, 700, 1363, 2344, and 7320 individuals. An additional core size of 10,000 was also studied to assess the changes in prediction accuracy when the core size is greater than the number of largest eigenvalues explaining over 99% of the variation in $\mathbf{G}$.

### Core definitions

#### Most popular animals (MPA)

This consisted of animals with a progeny size larger than a threshold (i.e., 400, 125, 35, 30, 20), with both parents known and own performance records. We considered MPA to test whether a core with stronger connections to other animals in the pedigree can result in a ${\mathbf{G}}_{APY}^{-1}$ that results in GEBV that are closer to those of ssGBLUP with direct inversion of $\mathbf{G}$.

#### Random set (Rnd)

Core animals were randomly sampled from all genotyped animals. This core definition was considered because previous studies demonstrated that sampling core animals at random would deliver identical prediction accuracy as ssGBLUP with direct inversion of $\mathbf{G}$, provided that the core size is equal to the number of eigenvalues explaining 98% of the variation in $\mathbf{G}$ [9, 10].

#### Uniformly distributed across pedigree (Ped)

Animals were sorted in genealogical order and then core animals were uniformly sampled across the pedigree. Since ${\mathbf{G}}_{APY}^{-1}$ is a sparse representation of ${\mathbf{G}}^{-1}$ [14, 16, 17], sampling animals across generations has the potential to capture all (co)variation in $\mathbf{G}$.

#### Within family selection (Fam)

Only one progeny within each full-sib family was randomly assigned to the core group. The data contained 43,367 full-sib families. This approach minimizes the relationships among core animals but ensures genetic ties between core and noncore animals.

#### Unrelated animals (Unrel)

Different genomic relationship thresholds were used (i.e., < 0.10, < 0.15, < 0.20, < 0.22, and < 0.27) to select unrelated individuals for the core group. If a pair of individuals had genomic relationships greater than the threshold, one was randomly excluded from the core. The aim of this core definition was to increase the genetic diversity in the core.

#### Least popular males (LPM)

Males with both parents known, without progeny, and without own performance records were included in the core. Considering that a GEBV is a combination of parent average, yield deviation, progeny contribution, and genomic information, animals with an own record or a progeny contribution already have an accurate EBV and consequently do not benefit much from genomic information. Hence, for this core definition, animals without progeny and own records were included in the core set and the remainder of genotyped animals were considered noncore.

#### Least popular females (LPF)

Females with both parents known, without progeny and own performance records were used as core. The justification behind this core definition is the same as for LPM.

#### QR decomposition (QR)

The gene content matrix ($\mathbf{Z}$) was decomposed into $\mathbf{Z}=\mathbf{Q}\mathbf{R}$ [22], where $\mathbf{Q}$ is an orthogonal matrix and $\mathbf{R}$ is an upper triangular matrix, and genotyped animals that correspond to the largest diagonal elements of $\mathbf{R}$ were selected as the core. The aim of this core definition was to select core animals with a high contribution to the (co)variation of $\mathbf{G}$, to enable faster convergence when solving the mixed model equations.

#### Regular ssGBLUP

Direct inversion of $\mathbf{G}$, i.e. all genotyped animals were included in the core, was used in ssGBLUP as the baseline for comparisons.

### Comparison of scenarios

#### Prediction accuracy

For each scenario, the accuracy of GEBV was calculated for young pigs born in 2020. On average, 17,594 genotyped pigs with phenotypes and born in 2020 were used as the validation set. The formula for computing the prediction accuracy was as follows:

$$\widehat{acc}=\frac{corr({\mathbf{y}}_{adj},\widehat{\mathbf{u}})}{h},$$

where ${\mathbf{y}}_{adj}$ is the vector of phenotypes adjusted for all non-genetic effects estimated using regular ssGBLUP, $\widehat{\mathbf{u}}$ is the vector of GEBV from ssGBLUP, and $h$ is the square root of the estimate of trait heritability. The mean squared error of prediction (MSE) was calculated as $MSE=\frac{1}{n}\sum_{k=1}^{n}{({\mathbf{y}}_{a\mathrm{dj},k}-{\widehat{\mathbf{u}}}_{k})}^{2}$, where $n$ is the number of animals in the validation set. The advantage of the MSE over other metrics is that it takes both variance and bias of the estimator into account.

#### Hierarchical clustering of GEBV

Dissimilarities of GEBV obtained using different core definitions were evaluated using a hierarchical clustering method. All core definitions were compared with a core size equal to the number of largest eigenvalues explaining 99% of the variation of $\mathbf{G}$ ($n$ = 7320). For each trait, a dissimilarity matrix of pairwise Euclidean norms between GEBV obtained from different core definitions was computed and entered into the “hclust” function in R [23] for clustering purpose. At each iteration of the hierarchical clustering method, we grouped the two most similar core definitions in order to identify hierarchical groups of core definitions with similar genomic signals, as captured by ssGBLUP. The distance between the newly merged group and each of the original core definitions was calculated by Ward’s criterion [24]. The idea follows Morota et al. [25], who studied dissimilarities between predictions from different genomic annotation models. However, our focus is on dissimilarities between GEBV from different core definitions in APY ssGBLUP.

#### Features of genomic relationships and allele frequency spectrum

Descriptive statistics of all elements and row sums of the absolute elements of the genomic relationship matrix between core and noncore animals (${\mathbf{G}}_{\mathrm{cn}}$) were investigated for a core size of 160 individuals, which equals the number of largest eigenvalues explaining 50% of the variation of $\mathbf{G}$. We hypothesized that core animals that have a higher contribution to the gene pool of the population show greater genomic relationships with noncore animals.

The overlap between minor allele frequency (MAF) spectrum of SNPs using core animals and the MAF spectrum for all genotyped individuals was investigated for each core definition and core size. For this purpose, SNPs were classified into five groups and coded as 1, 2, 3, 4 and 5 based on their MAF, i.e. 0–0.10, 0.10–0.20, 0.20–0.30, 0.30–0.40, and 0.40–0.50. Next, we quantified the proportion of overlap in MAF bins calculated using all genotyped individuals as $B$ against MAF bins calculated using $l$-th core set in each core definition at a given core size as ${C}_{l}$. Then, the proportion of overlap between $B$ and ${C}_{l}$ (overlap ($B$, ${C}_{l}$)) was calculated as follows:

$$overlap\left(B, {C}_{l}\right)=\frac{B\cap {C}_{l}}{m},$$

where $m$ denotes the total number of SNPs and $\cap$ is the intersection. A larger $overlap\left(B, {C}_{i}\right)$ corresponds to a greater chance of tracking ICS in the population based on animals in the $l$-th core.

## Results

Correlations of GEBV from regular ssGBLUP with those from APY ssGBLUP with each of the eight core definitions was greater than 0.99 when the core size equaled the number of largest eigenvalues explaining 99% of the variation in $\mathbf{G}$ (eigen99). For core definitions that distributed core animals across generations, i.e., Rnd, Ped, Fam, and Unrel, correlations for genotyped animals ranged from 0.994 to 0.997 (Table 2). For core definitions that did not prioritize across-generation distribution, i.e., MPA, LPM, LPF, and QR, correlations for genotyped animals ranged from 0.992 to 0.995. The smallest and largest numbers of iterations needed for APY ssGBLUP to reach the convergence criterion of 10^–12^ with each core definition at eigen99 were for the MPA and QR core definitions, respectively.

**Table 2:** Distribution of genotyped pigs across years for different core definitions when the core size was equal to the number of largest eigenvalues explaining 99% of variation in the genomic relationship matrix (n = 7320 animals)

Year	Total	Core definition							
		MPA	Rnd	Ped	Fam	Unrel	LPM	LPF	QR
2012	198	73	11	15	31	27	NA	NA	165
2013	866	312	47	54	134	85	1	1	510
2014	1731	749	108	118	275	137	3	2	918
2015	2968	922	204	205	442	309	1	1	1677
2016	7911	796	490	552	795	652	91	92	3444
2017	13,269	1766	866	926	1064	695	182	181	203
2018	27,766	1676	1819	1938	1667	1475	181	180	NA
2019	28,701	1014	1910	1915	1592	1565	1538	1538	NA
2020	27,184	12	1803	1529	1320	2163	5315	5317	NA
Sum^a^	110,594	7320	7258	7252	7320	7108	7312	7313	6917

MPA: popular animals with more than 15 progeny, both parents known and with own performance record; Rnd: a random subset of animals is sampled from all genotyped animals; Ped: animals were evenly selected from the pedigree sorted in genealogical order; Fam: from each full sib family, one progeny was allowed to be in the core group; Unrel: animals with $\le$ 0.27 genomic relationship were assigned to the core group; LPM: males with both parents known, without progeny, and without own performance records were used as core; LPF: females with both parents known, without progeny and own performance records were used as core; QR: animals selected based on a QR decomposition of the gene content matrix

^a^Some core definitions resulted in less than 7320 individuals because of missing birth year

**Fig. 1:**
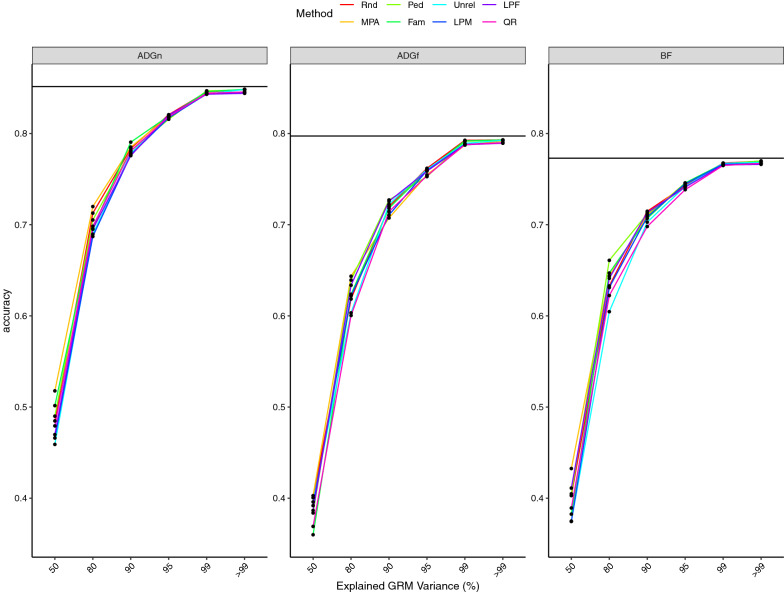
Prediction accuracy of genomic estimated breeding values from algorithm for proven and young single-step GBLUP (APY ssGBLUP) with different core definitions and core sizes for average daily gain in nursery (ADGn), average daily gain in finishing (ADGf), and backfat (BF). Core sizes were determined based on the number of largest eigenvalues explaining 50% (*n* = 160), 80% (*n* = 700), 90% (*n* = 1363), 95% (*n* = 2344), 99% (*n* = 7320) and > 99% (*n* = 10,000) of the genomic relationship matrix. The solid black line is the prediction accuracy estimated using regular ssGBLUP. MPA: most popular animals, both parents known and with own performance record; Rnd: a random subset of animals is sampled from all genotyped animals; Ped: animals were evenly selected from the pedigree sorted in genealogical order; Fam: from each full sib family, one progeny was allowed to be in the core group; Unrel: unrelated animals based on the genomic relationship were assigned to the core group; LPM: males with both parents known, without progeny, and without own performance records were used as core; LPF: females with both parents known, without progeny and own performance records were used as core; QR: animals selected based on a QR decomposition of the gene content matrix

**Fig. 2:**
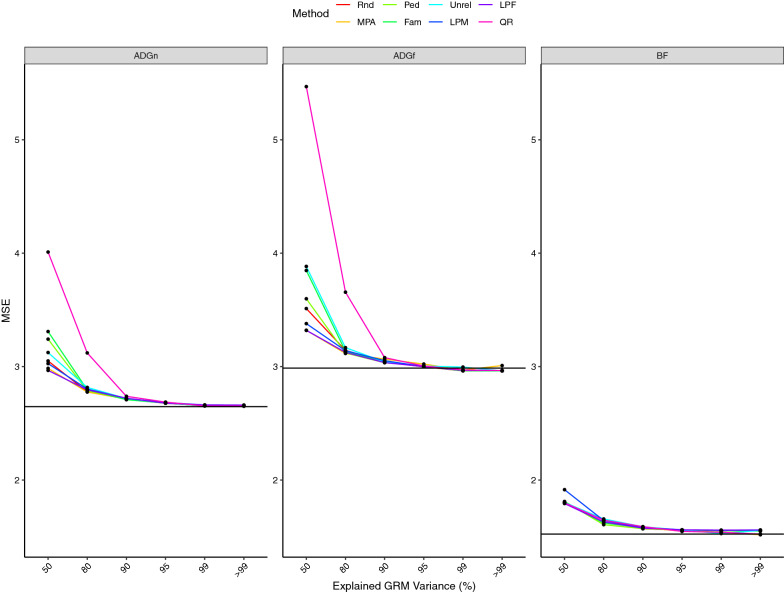
Mean squared error of genomic estimated breeding values from algorithm for proven and young single-step GBLUP (APY ssGBLUP) with different core definitions and core sizes for average daily gain in nursery (ADGn), average daily gain in finishing (ADGf), and backfat (BF). Core sizes determined based on the number of largest eigenvalues explaining 50% (*n* = 160), 80% (*n* = 700), 90% (*n* = 1363), 95% (*n* = 2344), 99% (*n* = 7320) and > 99% (*n* = 10,000) of the genomic relationship matrix. The solid black line is the prediction accuracy estimated using regular ssGBLUP. All symbols are defined in Fig. 1

The base line prediction accuracies from regular ssGBLUP were 0.85, 0.80, and 0.77 for ADGn, ADGf, and BF, respectively (Fig. 1) and the values of MSE for were 2.65, 2.97, and 1.53 for ADGn, ADGf, and BF, respectively (Fig. 2). When the number of animals in the core was equal to eigen99, all core definitions delivered similar prediction accuracies, slightly lower than the baseline (Fig. 1). In addition, at eigen99, all core definitions showed the same MSE as regular ssGBLUP (Fig. 2).

### Prediction accuracies with different core sizes and definitions

When the core size was less than the number of largest eigenvalues explaining 95% of the variation of $\mathbf{G}$ (i.e., eigen90), MPA delivered the highest prediction accuracy of all core definitions (Fig. 1). For this core size, QR and Unrel attained the lowest prediction accuracy. The results showed that differences in accuracy between core definitions disappeared as the core size increased, to nearly zero as the number of animals in the core set increased to the number of largest eigenvalues explaining 99% of the variation in $\mathbf{G}$.

Regardless of the trait and core definition, accuracy of APY increased by 47% from eigen50 to eigen99, by 18% from eigen80 to eigen99, by 8% from eigen90 to eigen99, and by 3% from eigen95 to eigen99. The improvement in the prediction accuracy when increasing the core size from eigen99 to 10,000 core animals was minimal (< 1%). When the core size was determined based on eigen99, the difference in prediction accuracy between APY ssGBLUP and regular ssGBLUP was less than 1% for three traits. For a core size of 10,000, differences in accuracy between regular ssGBLUP and APY scenarios were negligible.

For a core size of eigen50, prediction accuracies ranged from 0.46 (Unrel) to 0.52 (MPA) for ADGn, from 0.36 (Fam) to 0.40 (MPA, Rnd, and Unrel) for ADGn, and from 0.37 (Unrel) to 0.43 (MPA) for BF. However, as core size increased to eigen80, prediction accuracies ranged from 0.69 (Unrel, LPA) to 0.72 (MPA) for ADGn, from 0.60 (QR and Unrel) to 0.64 (MPA and Ped) for ADGf, and from 0.60 to 0.66 for BF. The difference in prediction accuracy among core definitions from eigen90 to eigen99 was less than 3%.

When increasing the core size from eigen50 to eigen99, MSE decreased dramatically (23%) (Fig. 2). Averaged across traits and core definitions, MSE declined by about 6% when core size increased from eigen80 to eigen99, by 2% from eigen90 to eigen99, and by 1% from eigen95 to eigen99. Increasing the core size beyond eigen99 did not change MSE significantly.

For a core size of eigen50, the difference in MSE between the best (MPA) and the worst core definition (QR) was 25% of one additive genetic standard deviation (SDa) for ADGn and 65% for ADGf. For BF at eigen50, LPF delivered the lowest MSE, and the other core definitions were similar in terms of MSE. As the core size increased to eigen80, differences in MSE between core definitions decreased, such that the difference in MSE between the core definition with the lowest (MPA) and that with the highest MSE (QR) was 12% SDa for ADGn and 16% SDa for ADGn. For a core size of eigen99, MSE was similar as for regular ssGBLUP.

### Hierarchical clustering of predicted genetic values

**Fig. 3:**
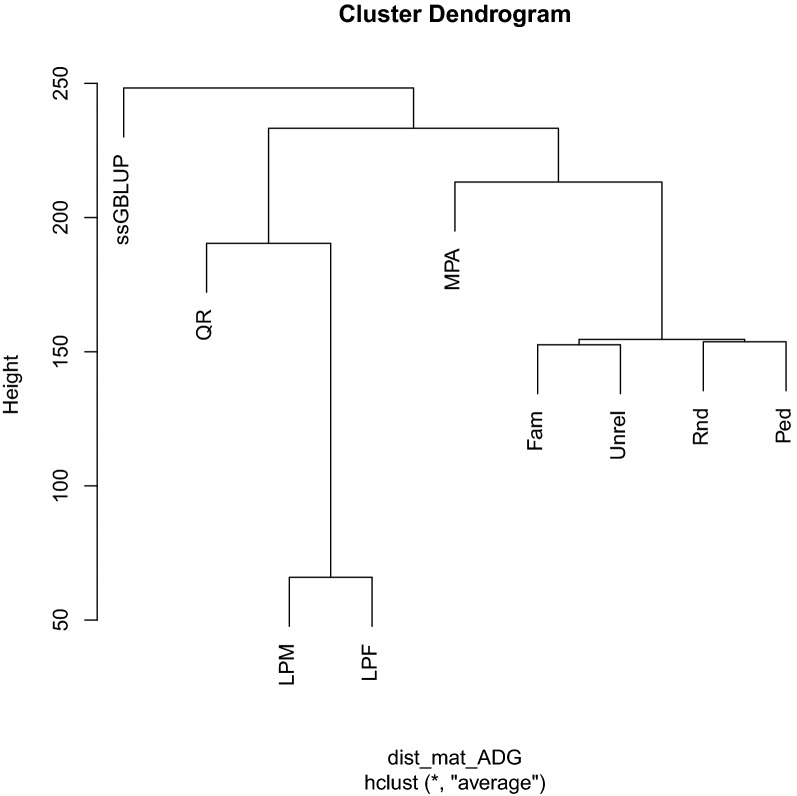
Hierarchical clustering of genomic estimated breeding values obtained from different core definitions for average daily gain in nursery. Core size was equal to the number of largest eigenvalues explaining 99% of the variation in the genomic relationship matrix. MPA: most popular animals, both parents known and with own performance record; Rnd: a random subset of animals is sampled from all genotyped animals; Ped: animals were evenly selected from the pedigree sorted in genealogical order; Fam: from each full sib family, one progeny was allowed to be in the core group; Unrel: unrelated animals based on the genomic relationship were assigned to the core group; LPM: males with both parents known, without progeny, and without own performance records were used as core; LPF: females with both parents known, without progeny and own performance records were used as core; QR: animals selected based on a QR decomposition of the gene content matrix

**Fig. 4:**
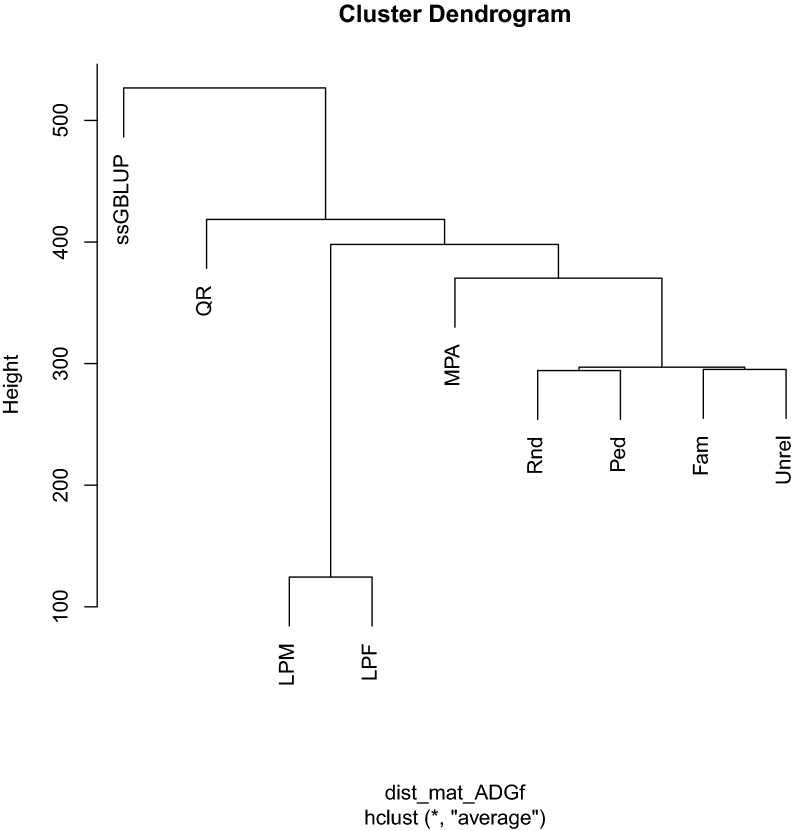
Hierarchical clustering of genomic estimated breeding values obtained from different core definitions for average daily gain in finishing. Core size was equal to the largest eigenvalues explaining 99% of variation in genomic relationship. All symbols are defined in Fig. 3

**Fig. 5:**
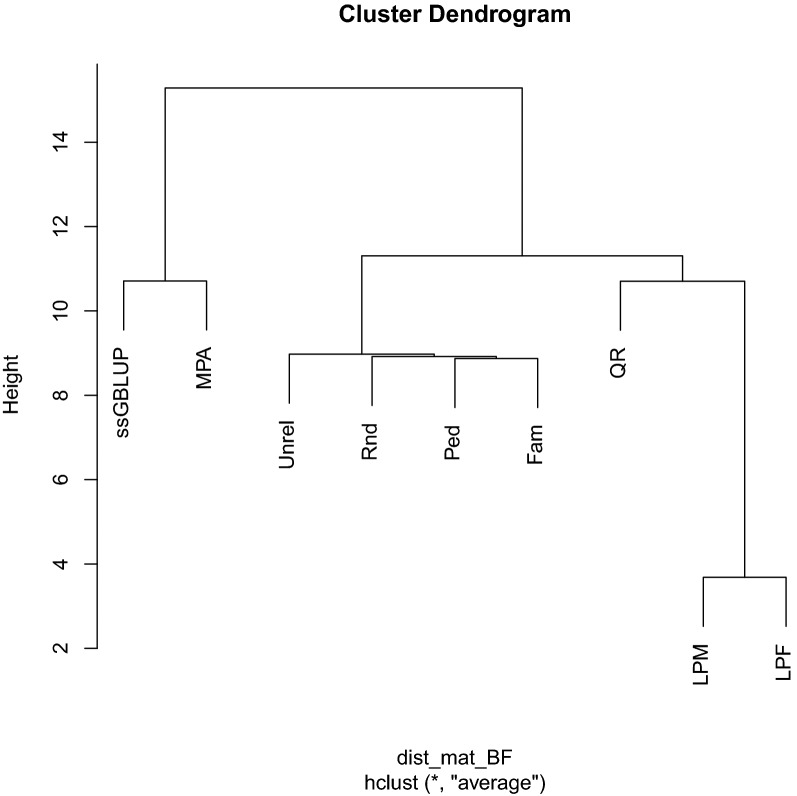
Hierarchical clustering of genomic estimated breeding values obtained from different core definitions for backfat. Core size was equal to the largest eigenvalues explaining 99% of the variation in the genomic relationship matrix. All symbols are defined in Fig. 3

Results of hierarchical clustering of GEBV obtained with a core size of eigen99 are shown in Figs. 3, 4, and 5. For both ADGn and ADGf, GEBV from regular ssGBLUP were at the top hierarchy on the dendrogram and GEBV from all other core definitions were its subdivisions.

Core definitions MPA and QR produced the most similar GEBV to those from ssGBLUP. The GEBV from Rnd, Ped, Fam, and Unrel were clustered in one group. The GEBV from LPM and LPF were most distant from the regular ssGBLUP. The trait BF had a slightly different dendrogram topology than ADGn and ADGf. For BF, GEBV from regular ssGBLUP and MPA were clustered together and GEBV from all other core definitions were gathered into a different cluster. Similar to ADGn and ADGf, GEBV from Rnd, Ped, Fam, and Unrel were grouped together and GEBV from LPM and LPF were classified far from regular ssGBLUP.

### Characteristics of genomic relationships between core and noncore animals

**Fig. 6:**
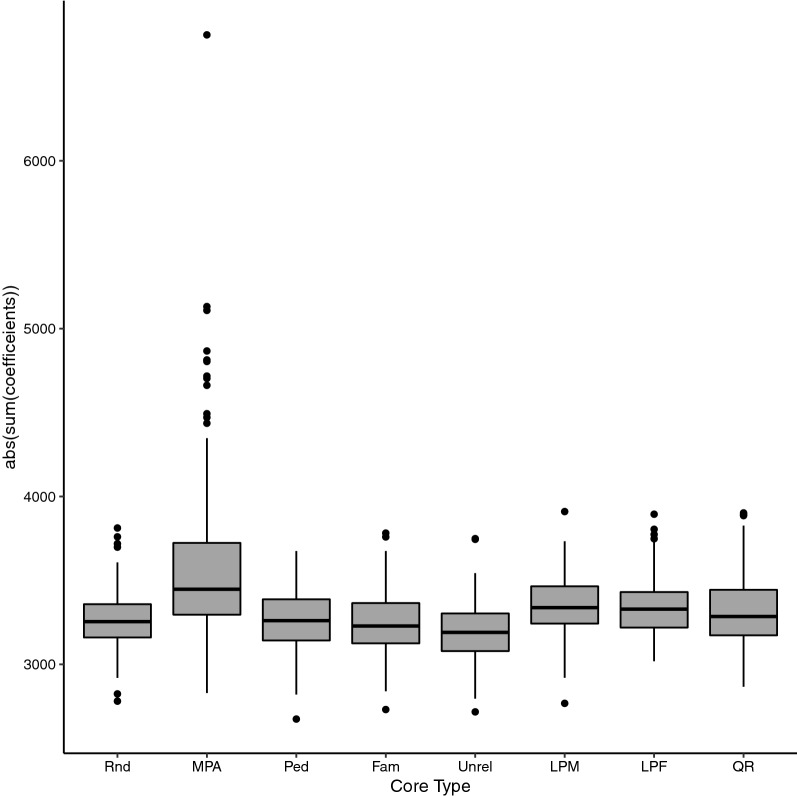
Distribution of the row sums of absolute elements of ${\mathbf{G}}_{\mathbf{c}\mathbf{n}}$ (genomic relationship between core and noncore animals) for different core definitions. Core size was equal to the number of largest eigenvalues explaining 50% of the variation in the genomic relationship matrix (*n* = 160). MPA: most popular animals, both parents known and with own performance record; Rnd: a random subset of animals is sampled from all genotyped animals; Ped: animals were evenly selected from the pedigree sorted in genealogical order; Fam: from each full sib family, one progeny was allowed to be in the core group; Unrel: unrelated animals based on the genomic relationship were assigned to the core group; LPM: males with both parents known, without progeny, and without own performance records were used as core; LPF: females with both parents known, without progeny and own performance records were used as core; QR: animals selected based on a QR decomposition of the gene content matrix

Row sums of the absolute elements of $\mathbf{G}$ between core and noncore animals (i.e., ${\mathbf{G}}_{\mathrm{cn}}$) for a core size of 160 (eigen50) is shown in Fig. 6. The results showed that, on average, MPA and Unrel resulted in the largest and smallest sum of rows in ${\mathbf{G}}_{\mathrm{cn}}$, respectively (Fig. 6). The mean ± SD of row sums of absolute elements in ${\mathbf{G}}_{\mathrm{cn}}$ was 3588 ± 514 for MPA and 3190 ± 182 for Unrel. The range of row sums of absolute elements in ${\mathbf{G}}_{\mathrm{cn}}$ was fourfold larger for MPA than for the other core definitions.

Overlaps of MAF spectra based on all individuals (= base line) and MAF spectra based on core animals are in Table 3 for each core definition and core size. In agreement with results for predictive ability and hierarchical clustering, the Rnd core definition had the largest overlap in MAF with the base line scenario, followed by Ped and Fam. In contrast, LPM, LPF, and QR showed the smallest overlap with the base line. As expected, increasing the core size from eigen50 to eigen99 increased the overlap in MAF spectra for core definitions with those of the baseline scenario.

**Table 3:** Proportion of overlap between minor allele frequency spectra calculated using all individuals and using core animals for each core definition and size

Core size	Core definition							
	MPA	Rnd	Ped	Fam	Unrel	LPM	LPF	QR
Eigen50	0.85	0.84	0.85	0.84	0.81	0.80	0.79	0.73
Eigen80	0.93	0.94	0.92	0.92	0.87	0.85	0.85	0.78
Eigen90	0.94	0.95	0.95	0.94	0.90	0.86	0.86	0.79
Eigen95	0.93	0.96	0.96	0.96	0.91	0.86	0.86	0.79
Eigen99	0.93	0.98	0.98	0.96	0.92	0.87	0.87	0.80
Eigen > 99	0.93	0.98	0.98	0.97	0.93	0.89	0.88	0.81

Eigen50, Eigen80, Eigen90, Eigen95, Eigen99 and Eigen > 99 denote core sizes equal to the largest number of eigenvalues explaining 50% (n = 160), 80% (n = 700), 90% (n = 1363), 95% (n = 2344), 99% (n = 7320) and > 99% (n = 10,000) of the genomic relationship matrix, respectively

MPA: popular animals with more than15 progeny, both parents known and with own performance record; Rnd: a random subset of animals is sampled from all genotyped animals; Ped: animals were evenly selected from the pedigree sorted in genealogical order; Fam: from each full sib family, one progeny was allowed to be in the core group; Unrel: animals with $\le$ 0.27 genomic relationship were assigned to the core group; LPM: males with both parents known, without progeny, and without own performance records were used as core; LPF: females with both parents known, without progeny and own performance records were used as core; QR: animals selected based on a QR decomposition of the gene content matrix

## Discussion

The APY approach computes a sparse representation of ${\mathbf{G}}^{-1}$ by dividing genotyped animals into core and noncore animals and ignoring relationships between noncore animals, which reduces computational costs. To thoroughly assess the effect of choice of the core on the prediction accuracy of GEBV from the APY approach, we investigated eight different core definitions, each one for six core sizes based on the number of largest eigenvalues explaining 50, 80, 90, 95, 99, and > 99% of the variation in $\mathbf{G}$. Therefore, this study may be the most comprehensive investigation of the effect of different core definitions and sizes on the performance of the APY approach.

### Core size

When the core size was smaller than the number of largest eigenvalues explaining 95% of the variation in $\mathbf{G}$, the difference in prediction accuracy between core types was more visible than when the core size was equal to or greater than eigen95. We found that the choice of core animals is important for the accurate prediction of GEBV using the APY approach when the core size is less than the number of largest eigenvalues explaining 95% of the variation in $\mathbf{G}$. However, as the core size increased to the number of largest eigenvalues explaining 98% or more of the variation in $\mathbf{G}$, all core definitions converged to a similar prediction accuracy and MSE. Pocrnic et al. [9] showed that the core size required to have as accurate GEBV as regular ssGBLUP is related to the dimensionality of $\mathbf{G}$, which depends on the number of ICS in the population, the number of SNPs, and the number of genotyped animals.

Pocrnic et al. [10] demonstrated that the most accurate GEBV based on APY ssGBLUP were obtained when the core size was equal to the number of largest eigenvalues explaining 98% of the variation in $\mathbf{G}$. In this regard, for simulated crossbreed pig populations, Vandenplas et al. [16] obtained the highest prediction accuracy when the core size was between the numbers of largest eigenvalues explaining 98% and 99% of the variation in $\mathbf{G}$. In a simulation study, Bradford et al. [15] reported that for a small core size (90% of the variation in $\mathbf{G}$), prediction accuracies of GEBV were lower than for regular ssGBLUP. Hence, a decrease in prediction accuracy is expected with small core sizes because some of the ICS are ignored in the calculation of ${\mathbf{G}}_{APY}^{-1}$. The increase in the overlap between MAF spectra based on all individuals and only core animals with increasing core size for a given core definition, indicates that the ability to track rare alleles improves as the number of core animals increases.

Assuming that the number of ICS in this study is around 8000, it is surprising that relatively high accuracies were obtained with 700 core animals (eigen80). Pocrnic et al. [26] found that the accuracy with $n$ randomly chosen core animals is almost as high as that considering only the $n$ largest eigenvalues in $\mathbf{G}$. They hypothesized that only a small number of core animals was required to identify clusters of the most popular chromosome segments in the population. Subsequently, the accuracy would be highest with the most popular animals, which was the case in our study when core size was small.

### Core definition

The correlation between GEBV obtained by regular ssGBLUP and by APY ssGBLUP with different core definitions at a core size of eigen99 was greater than 0.99. For the Rnd, Ped, Fam, and Unrel core definitions, the genotyped animals were well distributed across generations (Table 1). Rnd and Ped ensure across-generation representation of core animals. Therefore, when the core definition represents all generations, the likelihood that all ICS that segregate in the population are represented is high. Misztal [11] pointed out that the prediction performance of the APY approach depends on how well the core animals represent the ICS. The required number of core animals in APY to achieve the highest prediction accuracy is equal to the effective number of ICS, which can be computed as $\mathrm{ICS}=4{N}_{e}L$ [10, 11], where ${N}_{e}$ is the effective population size and $L$ the length of the genome in Morgans. Some studies have pointed out that core definitions that represent all generations and that maximize the number of genotyped offspring of core animals deliver the highest prediction accuracy [14, 16]. Including animals from all generations in the core set was also proposed by Bradford et al. [15].

Based on the clustering analyses, GEBV from MPA core were more similar to those from the regular ssGBLUP, followed by those from QR core. Fragomeni et al. [2] found that a core consisting of bulls with more than 10 daughters delivered a similar prediction accuracy as regular ssGBLUP. Ostersen et al. [14] reported that GEBV from APY ssGBLUP with core definitions involving random sampling of genotyped animals with large numbers of genotyped offspring had a high correlation with GEBV from regular ssGBLUP. They argued that when the core group represents a large proportion of ICS in the population, $\mathbf{G}$ constructed using this core has similar characteristics as the regular $\mathbf{G}$. However, the concept of ICS is hard to validate and deserves further research. Without costly computations of ICS/haplotype content of each core at each size, the proportion of SNPs with similar MAF based on a core set and based on all individuals can also indirectly provide some information about the possibility that the core captures the ICS in the population. As expected, the Rnd, Ped, and Fam core definitions showed the largest overlap between MAF spectra computed using all genotyped individuals and only those in the core due to an equal representation of genotyped animals across generations.

Although the prediction accuracy and MSE of GEBV for LPM and LPF at eigen99 were similar to those of other core definitions, the hierarchical clustering put the GEBV from these two core definitions in a separate group that was distant from that of the regular ssGBLUP. These two core definitions predominantly include young animals without progeny and phenotype records in the core and may, therefore, not capture a large proportion of the ICS segregating in the population because of recombination events that may have occurred across generations [15].

Regardless of the studied trait, the GEBV from APY ssGBLUP with the QR core at eigen99 were highly correlated with those from the regular ssGBLUP (0.992) but required the largest number of iterations to reach convergence. In contrast to the Rnd and Ped core definitions, the QR core included old animals born from 2012 to 2016. Hence, an unequal distribution of core animals across years or generations and a genetic lag between the core and recent genotyped populations is expected to result in poor convergence performance of APY. Furthermore, choosing old animals based on QR decomposition reduces the sparsity of ${\mathbf{H}}^{-1}$, possibly increasing the condition number and expanding the number of iterations to convergence. Thus, it is crucial to include animals from all generations in the core set.

Overall, we observed that the size of the core is more important than its definition. Sampling genotyped animals randomly from the available pool or evenly selecting animals across generations with the core size equal to the number of largest eigenvalues explaining 99% the variation in $\mathbf{G}$ provided accurate predictions of GEBV in this pig population. In multibreed or admixed populations with heterogeneous structure, the core definition may become more important. Mäntysaari et al. [27] observed that a core size greater than the number of largest eigenvalues explaining 98% of the variation in $\mathbf{G}$ was needed to achieve a high prediction accuracy in a multibreed beef cattle population. According to Cesarani et al. [28], more accurate predictions are obtained in multibreed evaluations when the core considers the dimensionality of $\mathbf{G}$ within each breed.

It is worth noting that the similarity of prediction accuracies for different core definitions can also be the result of the relationships between individuals in the core and noncore sets, provided that the core size is large enough to capture most of the (co)variation in $\mathbf{G}$. Fragomeni et al. [2] also found that a core of 20k young animals from a large pool of genotyped Holstein cattle resulted in a similar prediction accuracy than a core of proven bulls with more than five progeny. Although GEBV from APY and regular ssGBLUP have similar prediction accuracies and their correlations are higher than 0.99, individual GEBV may differ between core definitions. Concerns about changes in individual GEBV from changes in core definition and size can be alleviated by including selection candidates in the core [29].

## Conclusions

This study investigated the effect of core definition and size on the prediction accuracy of GEBV from APY ssGBLUP. When the core size is less than optimum, i.e. smaller than the number of largest eigenvalues explaining 98% to 99% of the variation in $\mathbf{G}$, the core definition is important. In such a case, core definitions for which core animals are well distributed across generations (i.e., most popular animals, randomly sampled, and evenly distributed across the pedigree) outperformed other core definitions in terms of prediction accuracy. However, as the core size reached or surpassed the optimum value, prediction accuracies were identical and the core definition became irrelevant.

## Data Availability

Provider of data does not intend to disclose its identity.
